# Myopenia and body fat distribution in hospitalized ulcerative colitis patients: correlations with clinical characteristics and response to vedolizumab

**DOI:** 10.3389/fnut.2024.1411695

**Published:** 2024-12-20

**Authors:** Wei Wei, Pengguang Yan, Yan Zhang, Qiong Wang, Junren Kang, Pengju Liu, Jin Fu, Jingnan Li, Kang Yu

**Affiliations:** ^1^Department of Clinical Nutrition, Peking Union Medical College Hospital, Chinese Academy of Medical Sciences and Peking Union Medical College, Beijing, China; ^2^Department of Gastroenterology, Peking Union Medical College Hospital, Chinese Academy of Medical Sciences and Peking Union Medical College, Beijing, China; ^3^Department of Radiology, Peking Union Medical College Hospital, Chinese Academy of Medical Sciences and Peking Union Medical College, Beijing, China

**Keywords:** myopenia, ulcerative colitis, adipose tissue, visceral obesity, vedolizumab

## Abstract

**Background:**

Ulcerative colitis (UC) patients often suffer from impaired nutritional conditions. However, there are few studies focused on muscle loss in UC patients as well as its impact on therapeutic response. This study aimed to investigate the prevalence of myopenia in hospitalized patients with active UC, analyze the relationship between body composition including both skeletal muscle and fat with clinical characteristics, and explore the association between body composition and clinical response to vedolizumab.

**Methods:**

A retrospective cohort study was conducted in hospitalized patients with active UC in Peking Union Medical College Hospital from November 2014 to October 2022. Computed tomography (CT) scans were used to measure skeletal muscle area, visceral fat area (VFA), subcutaneous fat area (SFA), and intramuscular fat infiltration at the third lumbar vertebrae (L3) level. These measurements were standardized by height (m) squared. Myopenia was defined as a skeletal muscle index (SMI) < 44.77 cm^2^/m^2^ for males and <32.50 cm^2^/m^2^ for females. The VFA/SFA ratio (VSR) served as an indicator of visceral obesity, while intramuscular fat infiltration was evaluated using the mean Hounsfield Unit (HU) value of the L3 skeletal muscle section.

**Results:**

A total of 457 patients were enrolled. The prevalence of myopenia was 49.7% in this cohort. Female patients had significantly higher levels of subcutaneous fat and intramuscular fat but a lower level of visceral fat than male patients. SMI and mean HU showed positive correlations with serum albumin (ALB) and negative correlations with serum high-sensitivity C-reactive protein (hsCRP), whereas VSR showed the opposite trend. Among the 92 patients who received vedolizumab treatment, myopenia was significantly associated with a lower clinical response rate, and this association remained significant after adjusting for vedolizumab duration, ALB, and hsCRP (OR = 3.458, 95% CI 1.238–9.659, *p* = 0.018). Visceral obesity, defined as VSR ≥ 75th centile of gender-specific VSR, tended to diminish the clinical response rate but did not reach statistical significance.

**Conclusion:**

This study underscores the significance of assessing body composition in UC patients. Optimizing body composition should be considered an integral component of managing UC patients in the future.

## 1 Introduction

Ulcerative colitis (UC) is one primary form of inflammatory bowel disease (IBD), characterized by chronic inflammation of the rectum and colon, which may lead to symptoms such as rectal bleeding, increased stool frequency, decreased stool consistency, and rectal urgency with a relapsing and remitting course (1). The standard treatment for mild to moderate UC typically involves oral 5-aminosalicylic acid. In cases of moderate to severe UC, biologics such as anti-TNF agents, anti-integrins, and anti-IL-12 and IL-23 have been recommended for inducing and maintaining remission (2). Myopenia is defined as clinically relevant muscle wasting that is associated either with impaired functional capacity and/or with an increased risk of morbidity or mortality (3). Over the past decade, few studies have paid attention to the prevalence of myopenia and its correlations with clinical parameters in UC patients.

The association between myopenia and prognosis has been explored in various diseases. For instance, preoperative myopenia has been identified as a negative predictive factor for cancer-specific survival and disease-free survival in patients undergoing colorectal cancer resection surgery (4). Additionally, myopenia correlates with radiographic joint damage in patients with rheumatoid arthritis (5). Moreover, in UC patients, myopenia is a risk factor for the need for surgical intervention, postoperative complications, and intravenous corticosteroid inefficacy (6). Vedolizumab, a recombinant humanized anti-α4β7-integrin monoclonal antibody, inhibits the migration of gut-homing memory T cells into the gastrointestinal submucosa, thereby reducing intestinal inflammation specifically (7). Vedolizumab has shown effectiveness in both inducing and maintaining remission in UC patients and has been increasingly used in the treatment of UC (8). However, data regarding the impact of muscle mass on response to vedolizumab in UC patients was sparse.

In addition to skeletal muscle, adipose tissue is another important component of body composition. It also gained some attention on its implications in disease prognosis, though less than muscle. Previous studies primarily focused on subcutaneous and visceral fat, with the latter predominantly comprising mesenteric and omental adipose tissues (9). Visceral adipose tissue can induce insulin resistance through proinflammatory cytokine and adipokine secretion, contributing to metabolic disorders (10). Subcutaneous adipose tissue depletion accelerates cachexia in cancer patients and leads to poor outcomes (11). Higher ratio of visceral fat area to subcutaneous fat area has been reported to be associated with higher possibility of undergoing surgery and higher frequency of disease flare in IBD patients (12, 13). However, adipose tissue distribution in UC patients and its correlation with therapeutic outcomes remain unclear.

This study aimed to investigate muscle mass loss and adipose tissue distribution in a cohort of patients with active UC, analyze the relationship between body composition and clinical data, and explore associations between body composition and response to vedolizumab therapy.

## 2 Materials and methods

### 2.1 Participants

Hospitalized patients aged 18 to 70 with active UC at Peking Union Medical College Hospital (PUMCH) were consecutively enrolled from November 2014 to October 2022. Inclusion criteria involved: (1) with complete medical records; (2) undergoing Computed tomography (CT) scans including the third lumbar vertebrae (L3) cross-section within a week before or after admission; (3) no UC surgery history; (4) had serum albumin (ALB) and high-sensitivity C-reactive protein (hsCRP) tests within a week before or after the CT examination mentioned above. Exclusions comprised comorbidities in major organs or auto-immune diseases beyond UC, spondyloarthropathy-related manifestations, neuromuscular or orthopedic issues impacting muscle health, cancer history, non-UC admissions, and pregnant or lactating females. For patients readmitted for UC recurrence or exacerbation, screening for inclusion/exclusion criteria was based on their initial admission. The study was approved by the Ethics Committee of PUMCH (No. I-22PJ700) and was conducted following the declaration of Helsinki.

### 2.2 Measurement of body composition by computed tomography

**Figure 1:**
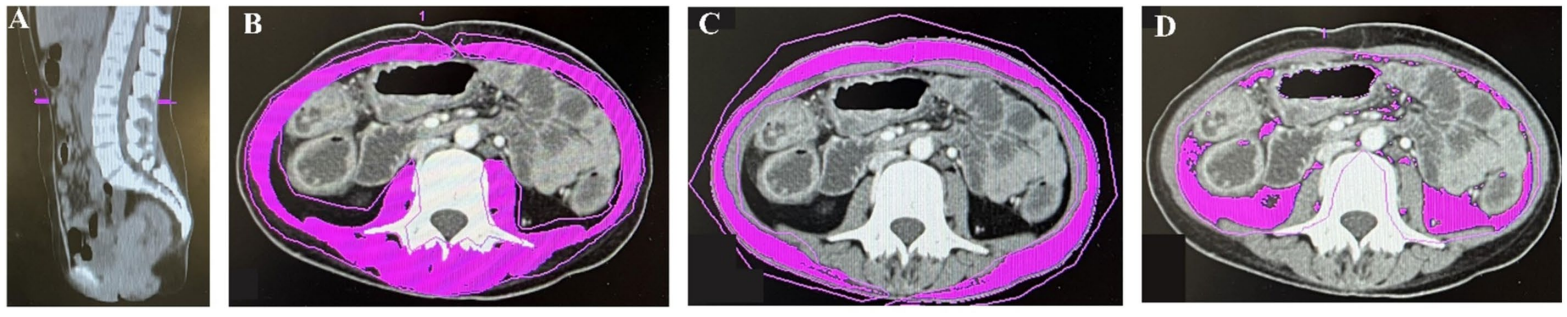
The midsagittal image in computed tomography (CT) was used to find the third lumbar vertebrae **(A)**. Skeletal muscle area (SMA) **(B)**, subcutaneous fat area (SFA) **(C)**, and visceral fat area (VFA) **(D)** were measured on CT images.

The CT data were obtained using the Picture Archiving and Communication System. The L3 muscle region has been demonstrated to best predict overall body composition (14). Skeletal muscle area (SMA), visceral fat area (VFA), and subcutaneous fat area (SFA) were quantified on L3 CT images using Syngo.via software (Siemens Healthineers, Forchheim, Germany) with specific Hounsfield Unit (HU) ranges for muscles (−29 to +150), subcutaneous adipose tissue (−190 to −30), and visceral adipose tissue (−150 to −50) (15) (Figure 1). SMA, VFA, and SFA values were normalized to height squared (m^2^), yielding skeletal muscle index (SMI), visceral fat index (VFI), and subcutaneous fat index (SFI) in cm^2^/m^2^, respectively. Myopenia was defined as SMI < 44.77 cm^2^/m^2^ in males and <32.50 cm^2^/m^2^ in females based on extensive research in China (16). Additionally, mean HU for L3 muscle assessed muscle quality, with lower values indicating greater fat infiltration, namely, myosteatosis (17). The VFA/SFA ratio (VSR) reflected the extent of visceral obesity.

### 2.3 Data collection

Demographic and clinical data including age, sex, body mass index (BMI), non-volitional weight loss, the percentage of food intake reduction, and disease duration were obtained from medical records. Disease activity was assessed following the modified Truelove and Witts’ criteria. Nutritional risk was evaluated using the Nutritional Risk Screening 2002 (NRS-2002), while malnutrition was diagnosed based on the Global Leadership Initiative on Malnutrition (GLIM) guidelines (18, 19). Results of serum ALB, hsCRP, and serum lipid tests [including triglyceride (TG), total cholesterol (TC), and low-density lipoprotein cholesterol (LDL-C)] within a week before or after the CT examination were collected.

### 2.4 Exploration of the correlation between myopenia and clinical response to vedolizumab

Patients treated with vedolizumab for induction and maintenance therapy over a minimum of 6 months, with CT scans containing L3 taken within 3 months before vedolizumab initiation, were included. The vedolizumab regimen consisted of 300 mg intravenously at weeks 0, 2, and 6, followed by doses every 8 weeks. Clinical response to vedolizumab was evaluated using the Mayo score for UC until the last vedolizumab administration before September 2023, with a positive response defined as a ≥3-point reduction in the Mayo score compared to baseline pre-vedolizumab (20).

### 2.5 Statistical analyses

Statistical analyses were performed using SPSS version 23.0 (SPSS Inc., Chicago, IL, United States) and GraphPad Prism version 6.0 (GraphPad Software Inc., San Diego, CA, United States). Continuous variables were expressed as mean ± standard deviation (SD) for normal distributions or median (Q1, Q3) for non-normally distributed data. Categorical variables were presented as numeric values (percentages). Group comparisons for continuous variables were performed using independent samples *t*-tests for normal distributions and the Mann–Whitney *U*-test for non-normal distributions. Categorical variable comparisons utilized the Chi-squared test, with significance set at *p* < 0.05. Pearson’s correlation or Spearman’s correlation was used to analyze associations between parameters based on the distribution characteristics of data. Multivariate logistic regression analysis identified risk factors for vedolizumab non-response, preceded by variance inflation factor calculations to check for collinearity among covariates (collinearity considered if variance inflation factor > 5).

## 3 Results

### 3.1 Prevalence of myopenia in UC patients

**Figure 2:**
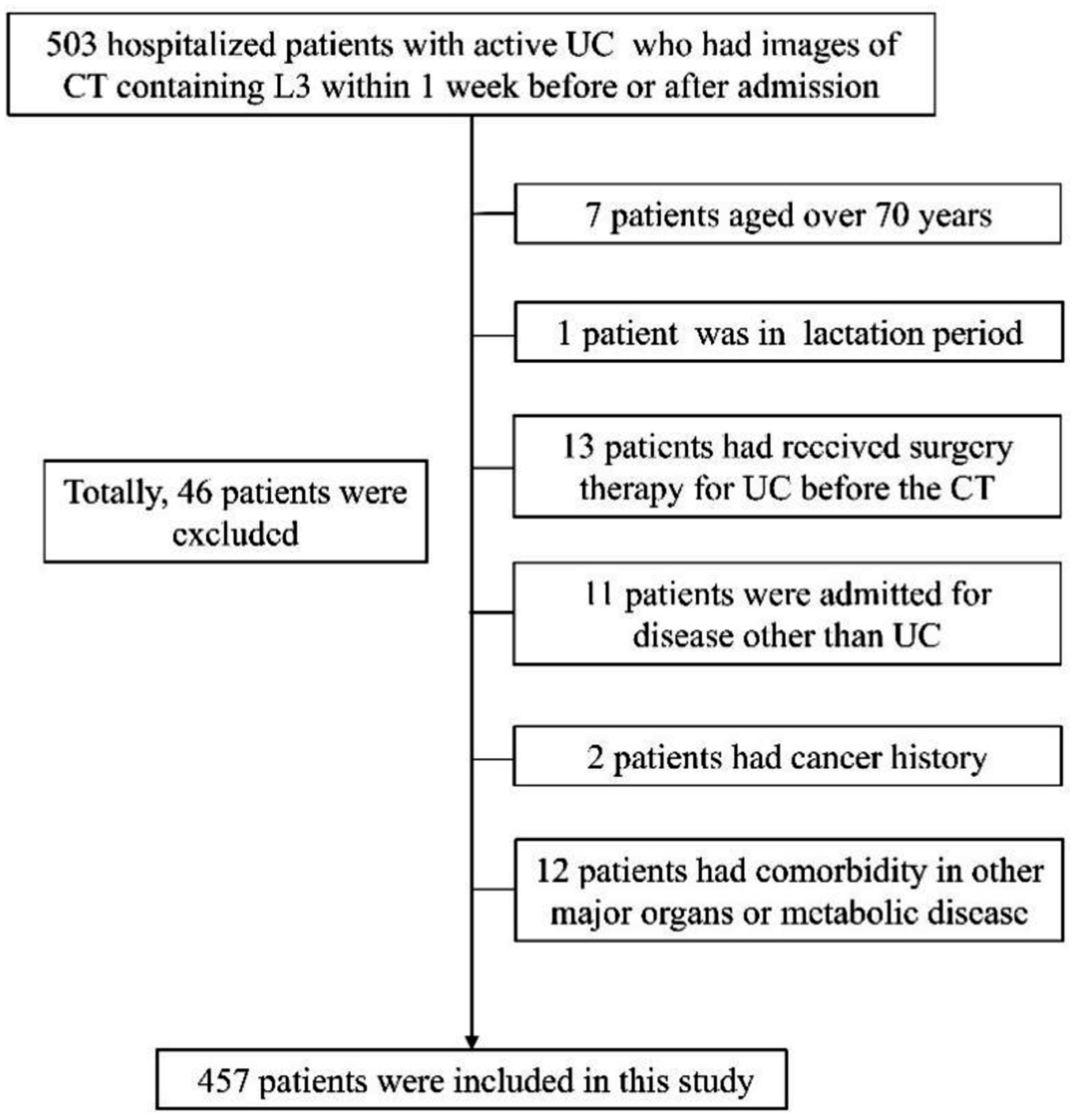
Flowchart for inclusion and exclusion of participants. Data collection timeframe was from November 2014 to October 2022.

A total of 457 hospitalized patients with active UC were analyzed, including 254 males and 203 females (Figure 2). Demographic, clinical, muscle, and fat indices data from CT scans are presented in Table 1. No significant differences were observed in age or disease duration between myopenic and non-myopenic patients. Myopenia prevalence was 49.7% (227 patients) in the overall cohort, with a slightly but not significantly higher rate in males (52.0%) than in females (46.8%). Myopenic patients had significantly lower VFA, VFI, SFA, and SFI values but a higher VSR than non-myopenic patients, denoting distinct fat distribution patterns in the two populations. Additionally, mean HU and ALB levels were significantly lower, while hsCRP levels were significantly higher in the myopenia group.

**Table 1:** The demographics, clinical characteristics, and body composition of ulcerative colitis patients with and without myopenia.

	Total (*n* = 457)	Myopenia (*n* = 227)	Non-myopenia (*n* = 230)	*p*^a^-value
Female (%)	203(44.4%)	95 (41.9%)	108 (47.0%)	0.272
Age, years	40.9 ± 12.7	41.0 ± 14.0	40.8 ± 11.2	0.834
Disease duration, months	48 (12, 96)	36 (12, 96)	48 (12, 96)	0.510
Disease activity				<0.001
Mild (%)	67 (14.7%)	23 (10.1%)	44 (19.1%)	
Moderate (%)	213 (46.6%)	93 (41.0%)	120 (52.2%)	
Severe (%)	177 (38.7%)	111 (48.9%)	66 (28.7%)	
BMI (kg/m^2^)	20.51 ± 3.30	18.98 ± 2.70	22.00 ± 3.16	<0.001
SMI (cm^2^/m^2^)	39.18 ± 8.23	34.13 ± 5.72	44.16 ± 7.23	<0.001
Mean HU	38.6 ± 7.5	36.9 ± 7.6	40.2 ± 7.1	<0.001
VFI (cm^2^/m^2^)	19.74 (9.66, 35.15)	16.17 (9.32, 31.40)	21.88 (9.94, 39.97)	0.022
SFI (cm^2^/m^2^)	30.88 (18.96, 46.17)	25.05 (15.45, 38.50)	35.84 (23.83, 51.06)	<0.001
VSR	0.64 (0.42, 1.02)	0.65 (0.47, 1.07)	0.63 (0.39, 0.94)	0.037
ALB (g/L)	34.5 ± 6.7	32.6 ± 6.8	36.4 ± 6.1	<0.001
hsCRP (mg/L)	12.03 (2.67, 39.86)	18.44 (5.12, 47.36)	7.50 (1.65, 28.34)	<0.001
Malnutrition	307 (67.2%)	197 (86.8%)	110 (47.8%)	<0.001

Data are presented as number (%), the median (Q1, Q3), or mean ± standard deviation.

a *p for differences between the myopenia group and the non-myopenia group.*

BMI, body mass index; SMI, skeletal muscle index; HU, Hounsfield Unit; VFI, visceral fat index; SFI, subcutaneous fat index; VSR, the ratio of the visceral fat area to the subcutaneous fat area; ALB, albumin; hsCRP, high-sensitivity C-reactive protein.

The prevalence of myopenia increased with disease activity, with proportions of 34.3, 43.7, and 62.7% in patients with mild, moderate, and severe disease activity, respectively. Myopenia was differently distributed between younger and older patients. In patients aged 18–50 years, the prevalence of myopenia was 44.9%, while in patients over 50 years, the prevalence of myopenia was 64.3% (*p* < 0.001). Among patients with BMI < 18.5 kg/m^2^ and ≥18.5 kg/m^2^, myopenia rates were 75.0 and 39.4%, respectively. Notably, within the myopenia subset, 56.4% had a BMI ≥ 18.5 kg/m^2^, while 4.0% had a BMI ≥ 24.0 kg/m^2^, with only one male patient surpassing a BMI of 28.0 kg/m^2^.

### 3.2 Association between myopenia and malnutrition

Among all the patients, 77.7% (355/457) were at nutritional risk according to NRS-2002, with 57.7% (205/355) of them exhibiting myopenia. Based on GLIM criteria, malnutrition prevalence was 67.2% (307/457). The rate of malnutrition was significantly higher in UC patients with myopenia compared with those without myopenia (86.8% vs. 47.8%, *p* ≤ 0.001, Table 1). No significant sex-based disparity was observed between malnourished and well-nourished groups (Table 2).

**Table 2:** The comparison of clinical characteristics and body composition in female and male ulcerative colitis patients.

	Female patients (*n* = 203)	Male patients (*n* = 254)	*p*-value
Age, years	40.8 ± 12.1	41.0 ± 13.1	0.868
Disease duration, months	48 (12, 108)	36 (12, 84)	0.362
Disease activity			0.334
Mild (%)	27 (13.3%)	40 (15.7%)	
Moderate (%)	93 (45.8%)	120 (47.3%)	
Severe (%)	83 (40.9%)	94 (37.0%)	
BMI (kg/m^2^)	19.85 ± 3.41	21.02 ± 3.12	<0.001
SMI (cm^2^/m^2^)	33.74 ± 5.53	43.52 ± 7.40	<0.001
Mean HU	35.8 ± 6.8	40.8 ± 7.3	<0.001
VFI (cm^2^/m^2^)	15.92 (9.64, 27.46)	22.69 (9.63, 41.58)	0.004
SFI (cm^2^/m^2^)	35.65 (23.59, 51.99)	26.70 (15.34, 39.63)	<0.001
VSR	0.48 (0.32, 0.67)	0.86 (0.56, 1.17)	<0.001
ALB (g/L)	33.8 ± 6.6	35.2 ± 6.8	0.017
hsCRP (mg/L)	11.98 (2.27, 42.57)	12.09 (3.27, 37.37)	0.646
Myopenia (%)	95 (46.8%)	132 (52.0%)	0.272
Malnutrition (%)	142 (70.0%)	165 (65.0%)	0.259

Data are presented as number (%), the median (Q1, Q3), or mean ± standard deviation.

BMI, body mass index; SMI, skeletal muscle index; HU, Hounsfield Unit; VFI, visceral fat index; SFI, subcutaneous fat index; VSR, the ratio of the visceral fat area to the subcutaneous fat area; ALB, albumin; hsCRP, high-sensitivity C-reactive protein.

### 3.3 Differences in body composition between male and female UC patients

The age, disease duration, disease activity, and serum hsCRP showed no significant difference between male and female patients. SFI was significantly higher in female patients, while BMI, VFI, VFR, and mean HU were all significantly lower in female patients (Table 2). Irrespective of sex, mean HU showed significantly negative correlations with SFI (*r* = −0.179, *p* = 0.011 in females, and *r* = −0.289, *p* < 0.001 in males), VFI (*r* = −0.443, *p* < 0.001 in females, and *r* = −0.510, *p* < 0.001 in males), and VSR (*r* = −0.421, *p* < 0.001 in females, and *r* = −0.508, *p* < 0.001 in males), especially with VFI and VSR.

### 3.4 Correlations between muscle or fat indices and laboratory indices in UC patients

**Figure 3:**
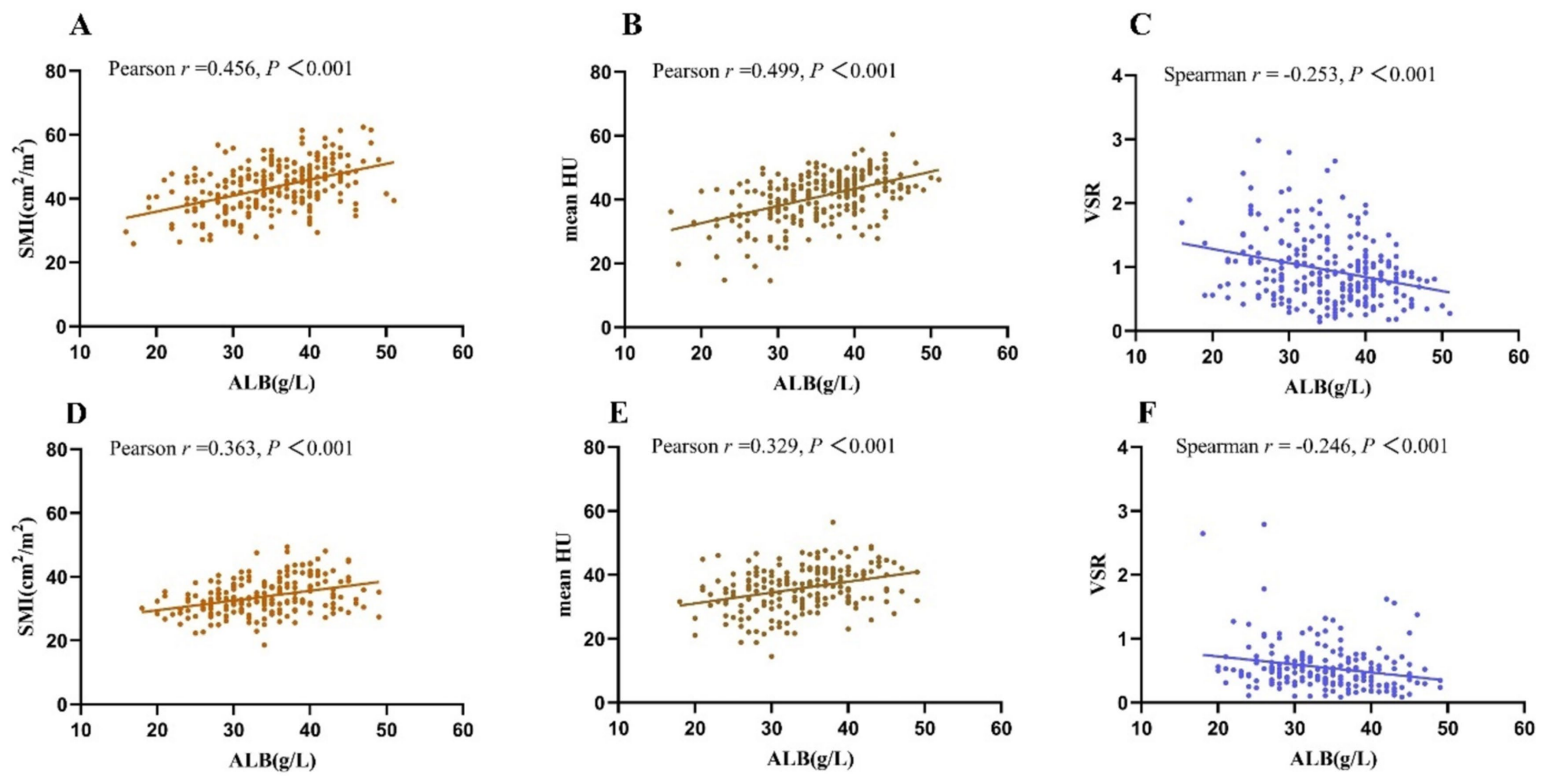
Both in female patients **(A–C)** and male patients **(D–F)**, SMI and mean HU showed significantly positive correlations with ALB, while VSR showed a significantly negative correlation with ALB. SMI, skeletal muscle index; VSR, the ratio of the visceral fat area to the subcutaneous fat area; HU, Hounsfield Unit; ALB, albumin; hsCRP, high-sensitivity C-reactive protein.

The SMI and mean HU showed significantly positive associations with ALB while VSR showed significantly negative associations with ALB, regardless of sex (Figure 3). SFI showed a significantly positive association with ALB only in female patients (*r* = 0.233, *p* = 0.001). Among the 222 patients with ALB levels <35 g/L, which is the cut-off value for hypoalbuminemia, 63.5% (141/222) had myopenia. Among the 235 patients with ALB levels ≥35 g/L, the prevalence of myopenia remained high at 36.6% (86/235).

Conversely, SMI and mean HU were negatively associated with hsCRP, and VSR showed a significantly positive association with hsCRP in female patients and a tendency of positive association with hsCRP in male patients (Table 3). Interestingly, hsCRP only showed a weak negative association with BMI in male patients (*r* = −0.129, *p* = 0.039) and showed no significant association with BMI in female patients.

**Table 3:** Correlations between muscle or fat indices and serum high-sensitivity C-reactive protein.

	Female		Male	
	*r*	*p-*value	*r*	*p-*value
SMI	−0.244	< 0.001	−0.287	<0.001
Mean HU	−0.220	0.002	−0.291	<0.001
VSR	0.190	0.007	0.108	0.086

Correlation was tested with Spearman’s correlation.

BMI, body mass index; SMI, skeletal muscle index; HU, Hounsfield Unit; VSR, the ratio of the visceral fat area to the subcutaneous fat area.

Subsequently, we explored the association between serum lipid levels and fat indices. Of all patients, 312 patients underwent serum lipid tests including TG, TC, and LDL-C within 1 week before or after CT. SFI showed a significantly positive association with TG. Moreover, a significant positive association between VFI and TG was observed (Table 4). There were positive associations between SFI or VFI with TC or LDL-C as well, and the associations with LDL-C were stronger than those with TC (Table 4). However, there were no significant associations found between mean HU or VSR with TG, TC, or LDL-C (data not shown).

**Table 4:** Correlations between fat indices and serum lipids.

	Female		Male	
	*r*	*p*-value	*r*	*p*-value
SFI vs.				
TG	0.169	0.044	0.208	0.007
TC	0.189	0.018	0.150	0.052
LDL-C	0.234	0.005	0.215	0.005
VFI vs.				
TG	0.229	0.006	0.339	<0.001
TC	0.152	0.069	0.157	0.042
LDL-C	0.163	0.053	0.204	0.008

Correlation was tested with Spearman’s correlation.

SFI, subcutaneous fat index; VFI, visceral fat index; TG, triglyceride; TC, total cholesterol; LDL-C, low-density lipoprotein cholesterol.

### 3.5 Correlations between body composition and clinical response to vedolizumab in UC patients

In our cohort, there were 92 patients (42 females and 50 males) who received vedolizumab and had CT images containing L3 cross-section within 3 months before the initiation of vedolizumab therapy. There were 67 patients with moderate disease activity and 25 patients with severe disease activity. The median therapy duration was 18 months, with an overall clinical response rate of 70.7% (65/92). When dividing these patients according to muscle mass, 36 patients had myopenia and 56 patients did not. The clinical response rate was significantly lower in the myopenia group [52.8% (19/36) vs. 82.1% (46/56), *p* = 0.003]. However, when comparing patients with a BMI < 18.5 kg/m^2^ to those with a BMI ≥ 18.5 kg/m^2^, no significant difference was observed in the clinical response rates (68.2% vs. 71.4%). Visceral obesity, defined as VSR ≥ 75th centile of sex-specific VSR in Table 2, was observed in 25 patients, and the clinical response rate showed a tendency to be lower in patients with visceral obesity but did not reach significance (60.0% vs. 74.6%, *p* = 0.170). Myosteatosis, defined as mean HU ≤ 25th centile of sex-specific mean HU in Table 2, was observed in 15 patients, with no significant difference observed in the clinical response rates between patients with and without myosteatosis.

Univariate analysis was performed on the baseline characteristics of the response group and non-response group (Table 5). The proportions of patients with malnutrition did not differ significantly between groups. Both groups showed no significant differences in age and sex distribution. Except for SMI, patients with and without clinical response to vedolizumab showed no significant difference in BMI or any of the other muscle and fat indices. Then, multivariate logistic regression showed that myopenia remained significantly associated with non-response to vedolizumab after adjusting for vedolizumab treatment duration, ALB, and hsCRP (OR = 3.458, 95% CI 1.238–9.659, *p* = 0.018).

**Table 5:** The baseline characteristics of ulcerative colitis patients receiving vedolizumab treatment.

	Response (*n* = 65)	Non-response (*n* = 27)	*p*-value
Female (%)	30 (46.2%)	12 (44.4%)	0.881
Age, years	41.8 ± 12.5	39.4 ± 9.9	0.381
Patients with severe disease activity (%)	16 (24.6%)	9 (33.3%)	0.392
VDZ treatment duration, months	22 (12, 29)	14 (8, 18)	<0.001
Myopenia (%)	19 (29.2%)	17 (63.0%)	0.003
Malnutrition (%)	30 (46.2%)	15 (55.6%)	0.411
BMI (kg/m^2^)	21.54 ± 3.58	20.65 ± 3.42	0.272
SMI (cm^2^/m^2^)	41.57 ± 8.80	37.74 ± 7.52	0.048
Mean HU	41.7 ± 7.5	39.9 ± 7.3	0.292
VFI (cm^2^/m^2^)	26.37 (11.95, 42.17)	27.45 (9.00, 44.30)	0.671
SFI (cm^2^/m^2^)	35.17 (25.74, 55.06)	34.48 (19.60, 47.89)	0.370
VSR	0.69 (0.41, 1.01)	0.61 (0.39, 1.26)	0.847
ALB (g/L)	38.0 ± 5.9	34.8 ± 7.2	0.026
hsCRP (mg/L)	2.71 (1.24, 9.10)	13.21 (4.60, 29.05)	0.005

Data are presented as number (%), the median (Q1, Q3), or mean ± standard deviation.

VDZ, vedolizumab; BMI, body mass index; SMI, skeletal muscle index; HU, Hounsfield Unit; VFI, visceral fat index; SFI, subcutaneous fat index; VSR, the ratio of the visceral fat area to the subcutaneous fat area; ALB, albumin; hsCRP, high-sensitivity C-reactive protein.

## 4 Discussion

This study provided some significant insights. The prevalence of myopenia was 49.7% among hospitalized patients with active UC, escalating to 64.3% in individuals aged over 50 years. Notably, 67.2% of patients were diagnosed with malnutrition according to GLIM criteria, close to our previous report (19). Among those with malnutrition, 64.2% had myopenia. This study is pioneering in its focus on sex variations in body composition based on CT scans in UC patients. It focuses on the relationship between body composition and clinical data in females and males, respectively. The indices reflecting muscle quantity and quality consistently showed positive associations with ALB levels and negative associations with hsCRP levels, whereas the parameter reflecting visceral fat accumulation showed an opposing trend. Moreover, this study described the relationship between baseline body composition and the clinical response to vedolizumab in UC patients, revealing myopenia as a potential predictor for poor response to vedolizumab.

Similar to our results, a recent single-center study involving 173 UC patients reported a myopenia prevalence of 53.2% (21). Zhang et al. (22) reported that the prevalence of myopenia in UC patients was 27.3%, but the sample size was relatively small (*n* = 99). The prevalence of myopenia spiked to over 60% in UC patients with severe disease activity in our cohort, consistent with reports indicating myopenia rates ranging from 50.2 to 69.5% among patients with acute severe UC (23, 24). Notably, over 50% of UC patients with myopenia had normal BMI levels in our cohort, consistent with earlier studies indicating that 51.2–60.1% of IBD patients with myopenia had a BMI ≥ 18.5 kg/m^2^ (25, 26). Furthermore, we found that only 4.0% of overweight or obese UC patients had myopenia, highlighting a lesser likelihood of myopenia occurrence in such patients despite probable weight loss during the disease course, whereas some previous studies reported that the rates could be as high as 12.6–19.5% (25, 26).

Although muscle mass has a crucial role in diagnosing malnutrition (18, 27), it is essential to note that low muscle mass does not always equate to malnutrition (28) since identifying nutritional risk stands as the primary step in diagnosing malnutrition, while our results showed that 42.3% of UC patients at nutritional risk had normal muscle mass. The relationship between muscle mass loss and malnutrition in UC patients has been sparsely explored. Given that nearly 40% of UC patients with a BMI ≥ 18.5 kg/m^2^ manifested myopenia on CT scans, evaluating muscle mass in UC patients is recommended to avoid overlooking those with poor nutritional status which can be hidden by a normal BMI. Our analysis showed that SMI had a stronger association with hsCRP compared to BMI in UC patients, hinting at inflammation’s greater impact on lean body mass loss than on body weight decline. Moreover, we found that myopenia, rather than low BMI before vedolizumab initiation, might increase the risk of treatment failure. A recent study on the association between low muscle mass and colectomy in acute severe UC patients also underscored myopenia, not low BMI, as a risk factor for rescue therapy and colectomy (26). Taken together, maintaining healthy muscle condition in UC patients may hold a stronger connection with disease severity and therapeutic effectiveness than normal weight, warranting increased attention in the management of UC in the future. Gastroenterologists and nutritionists can choose the measurement approach of muscle mass within their reach, like bioelectrical impedance analysis, Dual-energy X-ray absorptiometry, CT, and MRI.

Skeletal muscle, constituting about 40% of total body weight, represents the largest tissue in the human body (29). It undergoes dynamic changes due to factors like aging, illness, reduced physical activity, poor nutrition, and specific medications (3). Mitchell et al. reported a median muscle mass loss rate of 4.7% per decade in men and 3.7% per decade in women (30). As expected, we observed a remarkably higher prevalence of myopenia in UC patients over 50 years old compared to those aged between 18 and 50 years. However, it is noteworthy that in contrast with “inflammaging,” a term describing low-grade chronic systemic inflammation associated with aging (31), disease-induced inflammation can be more potent, serving as a primary trigger for rapid muscle mass depletion even in young patients (32, 33). Originating from colonic inflammatory conditions, UC triggers an upsurge in pro-inflammatory cytokines such as interleukins, TNF-*α*, and TGF-*β* (34). This systemic inflammation can inhibit the IGF-1/mTORC1 pathway, leading to increased protein catabolism and decreased muscle protein synthesis, recognized as a key mechanism for muscle wasting in UC (35, 36). Moreover, hypovitaminosis D, which is common in UC patients (37), deserves more attention in both systematic inflammation and myopenia in UC. Decreased 1,25-dihydroxycholecalciferol [1,25(OH)2D] and upregulated IL-33/IL-31 axis can alter the balance between inflammatory Th1/Th17 cells and T regulatory (Treg) cells and promote the bacterial translocation, associated with the pathophysiological processes of autoimmune diseases like IBD (38, 39). On the other hand, vitamin D insufficiency is correlated with reduced muscle function and sarcopenia (40). Meanwhile, skeletal muscle is acknowledged as a secretory organ capable of releasing myokines that counteract the detrimental effects of pro-inflammatory cytokines and alleviate inflammation (41). Our findings revealed a significant negative correlation between L3-SMI and serum hsCRP in UC patients, emphasizing the intricate but non-causal interaction between skeletal muscle and systematic inflammation.

In this study, we comprehensively investigated the links between subcutaneous fat, visceral fat, intramuscular fat, and systemic inflammation in active UC patients. We observed the positive associations between myosteatosis/visceral obesity and systematic inflammation in UC patients, as evinced by the negative correlation between mean HU and serum hsCRP as well as the positive correlation between VSR and serum hsCRP. Visceral and intramuscular fats have been shown to secrete an abundance of proinflammatory cytokines and cause the accumulation of proinflammatory immune cells (9, 42). Conversely, subcutaneous fat appears to have beneficial effects, evidenced by improved outcomes in gastric cancer patients with higher SFA and CD patients with increased SFI at the L3 level (43, 44). While we found no significant correlation between SFI and hsCRP, a positive correlation between SFI and ALB in female UC patients was observed in this cohort. Aligning with observations in healthy adults (45), our results demonstrated that both VFI and SFI positively correlated with serum TG, TC and LDL-C. Our team’s descriptive review proposed that blood lipids could be pivotal in fat redistribution, influencing the formation of visceral fat depots and fat infiltration in muscles and organs (42). The close relationship we noted between serum lipids and visceral fat bolsters this perspective. Interestingly, the higher serum TG level has been demonstrated to be associated with a higher possibility of surgery in UC patients (46, 47), which might be partly explained by the intimate connections among blood lipid level, body fat distribution, and systematic inflammation. Therefore, visceral and intramuscular fat may provide prognostic value to some degree in UC patients if they could be assessed regularly.

Similar to patients with cancer cachexia (48), we observed that UC patients with reduced muscle mass also had reduced subcutaneous fat and visceral fat. Systemic inflammation not only plays an crucial part in the waste of muscle and adipose tissue in UC patients, but also is of central importance in the mechanism of cachexia development (49). However, unlike in myopenic UC patients, the increase of serum hsCRP is not common in patients with cancer cachexia (50). Despite the reduction of both visceral and subcutaneous fat in myopenic UC patients, we found a higher VSR in this group. Considering higher systematic inflammation level (indicated by higher serum hsCRP) in myopenic UC patients, the stronger link between visceral fat and systemic inflammation than subcutaneous fat may be the critical factor leading to less waste of visceral fat than subcutaneous fat (9). Several studies have investigated the application of interventions that may improve muscle mass or muscle function in IBD patients. A study with a small sample size (*n* = 20) showed that 8-week moderate-intensity combined aerobic and resistance training could increase lean tissue mass and decrease fat mass in IBD patients (51). Moreover, 4-week resistance training together with whey protein was reported to significantly increase muscle mass in IBD patients when compared with resistance training and placebo (52). Collectively, structured exercise can decrease visceral fat mass and increase muscle mass, which has the potential to be an adjunctive therapy in IBD management (53).

A recently published article including 95 IBD patients reported that the total rate of clinical improvement after using vedolizumab was 74.8%, with an average duration of 17.83 months (54). Similarly, our cohort of UC patients treated with vedolizumab had a clinical response rate of 70.7% over a median duration of 18 months. Muscle loss can instigate a pro-inflammatory milieu due to deficient myokine signaling and compromised regenerative capabilities of immune cells (55), potentially leading to immunotherapy resistance. Therefore, better maintenance of skeletal muscle homeostasis may result in healthier immune functionality and a higher response rate to biologics. Recently, the relationship between visceral obesity and biologics treatment response in IBD patients has been explored in a few studies. A study including 68 IBD patients (with only 5 UC patients) reported that VSR was not correlated with treatment failure of anti-TNFα (56), while another study including 99 patients with Crohn’s disease (CD) found that a high VFI:SMI ratio was associated with an increased risk of failing standard doses of ustekinumab (57). A recently published study investigated the correlation between different biologics and endoscopic remission in IBD patient, including 141 patients in total (79 CD patients and 62 UC patients, with 52 patients using infliximab, 43 patients using ustekinumab, and 46 patients using vedolizumab), showing that patients (whether CD or UC patients) with higher visceral fat level were less likely to achieve endoscopic remission after the biologics treatment (58). In this study, we noted a lower albeit statistically insignificant clinical response rate in patients with visceral obesity, suggesting a potential adverse role of visceral obesity in inflammation regulation by vedolizumab in UC patients. Considering the important position of biologics in the therapy of IBD, the role of visceral obesity in the biologics response in IBD patients deserves further attention in the future.

There are several limitations in this study. Firstly, while CT scans offer an approach to evaluate muscle mass retrospectively, muscle strength and physical performance could not be assessed, precluding us from making the diagnosis of sarcopenia in these individuals. Secondly, it should be noted that UC patients who need to undergo CT examinations may have more severe disease manifestations than those who do not. Given that myopenia prevalence rises with disease severity, as exhibited in this study, the prevalence of myopenia might be overestimated. Similarly, in UC patients with milder disease activity (such as patients in Gastroenterology clinics), a lower prevalence of myopenia can be expected compared to the data in our cohort. Therefore, multi-center studies may help to enhance the representativeness of myopenia prevalence in hospitalized UC patients. Additionally, the results of the preliminary exploration of the relationship between body composition and vedolizumab response are incapable to determine causality, and need to be verified in a larger cohort in the future.

## 5 Conclusion

This study demonstrated that almost 50% of hospitalized patients with active UC had myopenia. Muscle quantity and quality at L3 showed a significant positive correlation with ALB and a negative correlation with hsCRP, while the degree of visceral obesity displayed an opposite pattern. Myopenia showed significant association with poor clinical response to vedolizumab. The assessment and optimization of body composition should receive more consideration in the future management of UC.

## Data Availability

The raw data supporting the conclusions of this article will be made available by the authors, without undue reservation.
